# Unraveling family resilience patterns in ICU first-episode stroke: a latent profile analysis

**DOI:** 10.3389/fpsyg.2026.1673403

**Published:** 2026-01-26

**Authors:** Jinlei Du, Jin Yang, Yulian Wu, Ling Lei, Chencong Nie, Xiaoling Wu

**Affiliations:** Zigong Fourth People’s Hospital, Zigong, China

**Keywords:** family resilience, ICU, influencing factors, latent profile analysis, stroke

## Abstract

**Objective:**

To identify latent family resilience profiles among families of patients with first-episode stroke in the intensive care unit (ICU) and examine factors associated with resilience heterogeneity, with the aim of informing targeted family-support interventions.

**Methods:**

A cross-sectional study was conducted among 335 ICU patients with first-episode stroke and their primary caregivers. Family resilience was assessed using the Chinese version of the Family Resilience Assessment Scale (FRAS-C). Latent profile analysis (LPA) was used to identify subgroups of family resilience, while LASSO regression and multiple binary logistic regression were applied to determine influencing factors.

**Results:**

Two distinct resilience profiles were identified: Developing Families, characterized by lower levels of communication, resource utilization, and positive outlook; and Optimized Families, characterized by higher resilience across all dimensions. ICU admission count (OR = 2.299, 95% CI: 1.066–4.960), frequency of care and support from relatives or friends (OR = 1.851, 95% CI: 1.068–3.206), and number of additional organ system dysfunctions (OR = 0.233, 95% CI: 0.122–0.445) were significantly associated with family resilience profiles (all *P* < 0.05).

**Conclusion:**

Family resilience among ICU first-episode stroke patients shows notable heterogeneity, with two typical resilience patterns. Early identification of high-risk families—particularly those with limited social support or higher disease complexity—can guide clinicians in delivering targeted communication support, psychological counseling, and resource linkage interventions. Tailored resilience-enhancing strategies may contribute to better patient recovery and improved family adaptation during critical care.

## 1 Introduction

Stroke is one of the leading causes of adult disability and mortality worldwide (Wei et al., 2025). Characterized by abrupt onset and rapid progression, it often poses a critical threat to patients’ lives within a short period of time. In China, stroke has become one of the most common chronic diseases, and the 2021 China Stroke Surveillance Report indicates that 3.4 million first-ever stroke cases occurred in 2020 alone, with the burden continuing to rise (Tu and Wang, 2023). During the acute phase of stroke, patients often present with impaired consciousness, hemiplegia, speech disorders, or even cardiac arrest, requiring intensive monitoring and advanced life support in the intensive care unit (ICU) (de Montmollin et al., 2020).

Recent studies further indicate that the pathophysiology of acute stroke is strongly influenced by systemic inflammatory responses and stress-induced hyperglycemia (SIH) (Cai et al., 2023). Inflammation contributes to secondary brain injury, disrupts the blood–brain barrier, and amplifies neurological impairment, while SIH—common among critically ill ICU patients—has been identified as an independent predictor of poor neurological recovery and adverse outcomes (Shen et al., 2024). These mechanisms not only worsen the clinical severity of stroke but also increase treatment uncertainty, extend ICU care needs, and intensify the psychological and caregiving burden experienced by family members. Consequently, the acute onset of stroke, coupled with inflammation- and stress-related physiological instability, places families under greater emotional strain and highlights the need to understand how different families adapt to such high-intensity stressors, particularly within the ICU environment.

As a highly specialized and isolated care setting, the ICU imposes strict visitation policies that, while safeguarding treatment outcomes, severely restrict family presence and engagement (Tronstad et al., 2021). This “informational disconnection” and “emotional absence” significantly heighten family members’ anxiety, helplessness, and decision-making stress, thereby compromising their psychological wellbeing and quality of life. At the same time, family members are placed into a high-stress state, facing intense emotional strain and urgent medical decisions (Hayes et al., 2024). Families experiencing a patient’s first-ever stroke face a particularly abrupt and unfamiliar crisis, as they must rapidly adapt to critical care routines, limited communication with the patient, and high medical uncertainty—all of which compound psychological stress and disrupt family functioning. Against this backdrop, family resilience, defined as the collective ability to maintain function and adapt under stress, becomes pivotal for patient recovery and caregiver adjustment.

Family resilience refers to a family’s ability to recover, adapt, and even grow in the face of adversity, stress, or major life disruptions, relying on internal cohesion, external support, and a shared belief system (Nadrowska et al., 2022). Walsh’s family resilience framework conceptualizes it across three core dimensions: belief systems, organizational patterns, and communication/problem-solving processes (Walsh, 2003). Studies have shown that high levels of family resilience not only promote psychological wellbeing among family members but also provide essential emotional and social support for patient recovery (Shao et al., 2023). While existing research on family resilience has primarily focused on community populations or individuals with chronic illnesses (Qiu et al., 2025), limited attention has been given to families in ICU settings, which are marked by high stress and uncertainty. In particular, families facing a patient’s first-time ICU admission due to stroke often encounter acute psychological distress and role disruption. The variation in family resilience in this context not only influences the quality of support provided to the patient but also affects the family’s own psychological adjustment and systemic stability.

Therefore, identifying the latent profiles and influencing factors of family resilience in ICU patients with first-episode stroke serves both theoretical and practical purposes. It fills a gap in the critical care literature by incorporating the “family dimension” and offers an empirical basis for stratified identification of high-risk families and personalized intervention strategies. Given that family resilience is a multidimensional construct with potentially diverse underlying patterns, latent profile analysis (LPA) is particularly suitable for identifying unobserved heterogeneity and classifying families into distinct subgroups based on resilience characteristics. Accordingly, this study employed latent profile analysis (LPA) to explore the heterogeneity of family resilience in this population and further examined its key determinants, with the aim of providing theoretical and practical guidance for optimizing nursing care pathways and enhancing the effectiveness of family support.

## 2 Materials and methods

### 2.1 Study participants

Between June 1 and June 30, 2025, we conducted convenience sampling in 55 tertiary-care ICUs located in eight administrative regions of central and western China, including Sichuan, Shaanxi, Guizhou, Yunnan, Qinghai, Gansu, Guangxi Zhuang Autonomous Region, and Chongqing. Convenience sampling was adopted due to the wide geographic distribution and time constraints across multiple tertiary hospitals. While this approach may limit strict representativeness, it ensured feasibility and timely data collection in a multi-center context. Random sampling was not feasible in this multicenter ICU study because of differences in patient admission timing, the urgent nature of critical illness, and ethical constraints related to patient and caregiver participation. The study population comprised patients with first-episode stroke admitted to the ICU during the study period and their primary family caregivers. Each patient was matched with one primary family caregiver to ensure consistency in reporting and analysis.

The sample size was initially estimated based on the precision requirement for the mean FRAS-C score, with an allowable error of ± 1 unit; considering a potential 10% invalid response rate, the planned target was 238 participants. Because the main analysis involved multiple binary logistic regression, the adequacy of the final sample was further evaluated using the events-per-variable (EPV) criterion, which recommends approximately 10 outcome events per predictor to ensure model stability (Peduzzi et al., 1996). In the present study, the final multiple binary logistic regression model included three predictors, and the smaller outcome category comprised 45 participants, yielding an EPV of approximately 15 (45/3). This exceeds the commonly accepted threshold and suggests that the model is adequately powered and stable. Ultimately, a total of 335 valid samples were obtained, exceeding the theoretical sample size.

Inclusion criteria for patients were as follows: (1) Stroke diagnosis was based on the American Heart Association/American Stroke Association (AHA/ASA) clinical guidelines for acute stroke, requiring neuroimaging confirmation (CT or MRI) to differentiate ischemic from hemorrhagic events (Choi et al., 2022).“First-episode stroke” was defined as the first-ever clinically diagnosed and imaging-confirmed ischemic or hemorrhagic stroke, with no previous history of stroke; (2) ICU stay duration ≥ 24 h;(3) Informed consent obtained from the patient or their family, with voluntary participation in the study. Exclusion criteria for patients included: (1) Patients with any previous imaging-confirmed stroke were excluded; (2) Death within 48 h of ICU admission; (3) Transfer to another hospital or voluntary withdrawal from treatment during the course of care. Inclusion criteria for primary family caregivers were as follows: (1) A direct biological relationship with the patient (e.g., parent, sibling, or child); (2) Informed consent and voluntary participation in the study. Exclusion criteria for primary family caregivers included: (1) Presence of speech or language disorders; (2) Participation in other psychological research studies within the past 3 months. During the study, if either the patient or their primary caregiver declined or withdrew from participation, data from both parties were excluded from the analysis.

### 2.2 Ethics approval and consent to participate

This study was approved by the Ethics Committee of Zigong Fourth People’s Hospital (approval number: 2025-036) and conforms to the National Health and Medical Research Council, Australian Research Council, & Universities Australia (2018). The study was conducted in accordance with the Declaration of Helsinki. All participants provided written informed consent to participate in the study. All interviews and surveys were conducted in private settings to ensure participant comfort and privacy. Participants were informed that their participation was entirely voluntary and that they could withdraw from the study at any time without any consequences. All collected information was processed anonymously to protect participant privacy.

### 2.3 Instruments

#### 2.3.1 General information questionnaire

The general information questionnaire was developed by the research team based on the clinical experience and a review of relevant literature. A preliminary draft was formulated through group discussions and finalized through expert consultation with five specialists in the field. The questionnaire consisted of two sections: patient-related information and caregiver-related information.

The patient section included data such as gender, age, occupational status, marital status, educational level, Acute Physiology and Chronic Health Evaluation II (APACHE II) score, and stroke type. Marital status was classified as “married” (currently in a marital relationship) or “unmarried” (including never married, divorced, or widowed). For stroke type, the “other” category referred to conditions such as cerebral venous thrombosis or cerebral amyloid angiopathy. The primary caregiver section collected information including age, gender, educational level, medical expense payment, understanding of the disease and other relevant background characteristics.

#### 2.3.2 Chinese version of the Family Resilience Assessment Scale

The original Family Resilience Assessment Scale (FRAS) was developed by Sixbey in 2005 based on Walsh’s family resilience framework (Sixbey, 2008). In 2016, Li Yuli et al. translated and culturally adapted the scale for Chinese populations, resulting in the Chinese version (FRAS-C) (Li et al., 2016). The scale comprises 32 items across three dimensions: Family Communication and Problem Solving (FCPS),Utilization of Social Resources (USR), and Maintaining a Positive Outlook (MPO). Each item is rated on a 4-point Likert scale ranging from 1 (“strongly disagree”) to 4 (“strongly agree”), with a total score ranging from 32 to 128. Higher scores indicate higher levels of family resilience. The scale demonstrated excellent internal consistency, with a Cronbach’s alpha coefficient of 0.95 in the original version and 0.94 in this study.

### 2.4 Data collection

Data were collected through paper-based questionnaires distributed to participating medical institutions via express delivery or local distribution. Prior to data collection, the research team engaged in thorough communication with ICU healthcare staff across participating institutions, providing detailed explanations of the study’s background, objectives, and data collection procedures. Trained investigators introduced the study purpose and questionnaire completion instructions to primary family caregivers once their emotional state was relatively stable. Upon obtaining informed consent, questionnaires were administered. The questionnaire comprised two sections: Patient-related information, which was extracted from medical records using hospital information systems; and Caregiver-related information, which was completed on-site by the family caregivers themselves.

During the completion of the questionnaire, trained researchers provided real-time guidance to ensure accurate understanding and truthful responses. All completed questionnaires were collected immediately upon completion. For caregivers with lower literacy levels, a face-to-face interview approach was adopted, with researchers filling out the questionnaire based on the caregiver’s verbal responses. Completed questionnaires were then collected and mailed back to the research team by institutional coordinators.

### 2.5 Quality control

A dedicated research team was established to oversee quality control throughout the study. Each participating institution appointed a designated coordinator responsible for site-level quality management. Before the investigation began, all research staff and site coordinators received standardized training covering study objectives, questionnaire content, and data collection techniques. Only those who passed a post-training assessment were authorized to participate in data collection.

After the survey phase, the research team conducted random sampling audits of questionnaires from each institution. If three or more questionnaires from the same institution were found to be non-compliant, all questionnaires from that site were excluded from final data analysis. During the data entry phase, two graduate students with medical backgrounds independently double-entered the data. Questionnaires with illegible handwriting or excessive corrections were excluded. Additionally, all statistical analyses and result interpretations were reviewed by experts in biostatistics to ensure scientific rigor and clinical relevance.

### 2.6 Statistical analysis

Exploratory Latent Profile Analysis (LPA) was conducted using Mplus version 8.3 to identify the optimal latent profile structure of family resilience, based on the three dimensions of the Chinese version of the Family Resilience Assessment Scale (FRAS-C). One- to five-class models were fitted. Model fit was evaluated using the Akaike Information Criterion (AIC), Bayesian Information Criterion (BIC), adjusted Bayesian Information Criterion (aBIC), and entropy value s. Lower AIC, BIC and aBIC indicate superior fit. Entropy values closer to 1.0 suggest a higher classification accuracy; entropy values around 0.8 typically correspond to classification accuracy exceeding 90% (Du et al., 2025b).

In addition, to enhance the precision of variable selection, the Least Absolute Shrinkage and Selection Operator (LASSO) regression was employed to preliminarily screen potential influencing factors. The regularization parameter λ was determined via 10-fold cross-validation, with the optimal λ selected as the value that minimized the mean cross-validated error. By applying an L1 regularization penalty to the regression coefficients, LASSO enables both variable selection and model shrinkage. This approach effectively mitigates multicollinearity among variables, thereby improving model robustness and generalizability. Variables with non-zero coefficients identified by LASSO were considered potentially predictive and included in the binary logistic regression model for further analysis, as this combination approach effectively addresses multicollinearity among numerous predictors and enhances the robustness of variable selection and model reliability.

Prior to logistic regression modeling, multicollinearity diagnostics were performed on all candidate variables. The Variance Inflation Factor (VIF) was used to assess inter-variable correlation, with VIF > 10 indicating severe multicollinearity. The Hosmer-Lemeshow goodness-of-fit test was used to assess the adequacy of the logistic regression model, with *P* > 0.05 indicating good model fit—that is, predicted outcomes aligned well with observed results. Regarding missing data, no missing values were present in the final dataset used for analysis; all questionnaires were completed in full and checked for completeness prior to data entry. All statistical tests were two-tailed, and *P* < 0.05 were considered statistically significant.

All statistical analyses were performed using Mplus version 8.3 for latent profile analysis, IBM SPSS Statistics version 26.0 for logistic regression, and R (version 4.3.2) with the glmnet package for LASSO regression.

## 3 Results

### 3.1 General information of the participants

A total of 400 questionnaires were distributed in this study, and 372 were returned. Among them, 10 questionnaires were excluded due to repeated modifications, and 7 were excluded due to illegible handwriting. Ultimately, 335 valid questionnaires were obtained, resulting in a valid response rate of 95.43%. To reduce sampling error and ensure the robustness of the findings, all 335 valid samples were included in the final analysis. Among the 335 first-episode stroke patients admitted to the ICU, 174 were male and 181 were female, with a mean age of 46.10 ± 16.24 years. Regarding the 335 primary family caregivers, 211 were male and 144 were female, with a mean age of 48.32 ± 12.37 years. The total score on the Chinese version of the Family Resilience Assessment Scale was 92.88 ± 14.66. The scores for each dimension were as follows: family communication and problem solving, 67.74 ± 10.92; utilization of social resources, 7.80 ± 1.98; and maintaining a positive outlook, 17.33 ± 3.56.

### 3.2 Latent profile analysis of family resilience in first-episode stroke patients

Based on the dimensional scores of the Chinese version of the Family Resilience Assessment Scale, five latent profile models were constructed to explore the heterogeneity of family resilience among first-episode stroke patients in the ICU. Model fit was comprehensively evaluated using the Akaike Information Criterion (AIC), Bayesian Information Criterion (BIC), adjusted BIC (aBIC), Entropy, Lo-Mendell-Rubin (LMR) test, and Bootstrap Likelihood Ratio Test (BLRT). Entropy values closer to 1.0 indicate clearer classification, with values above 0.8 generally corresponding to classification accuracy exceeding 90%, and values above 0.9 indicating excellent classification precision. As shown in Table 1, with the increase in the number of latent classes, the AIC, BIC, and aBIC values generally showed a decreasing trend. When the number of classes reached two, the Entropy value was the highest (0.956, indicating excellent classification accuracy), and both the LMR and BLRT tests indicated statistically significant differences (*P* < 0.05), suggesting that the two-class model had the best fit.

**TABLE 1:** Model fit indices for latent profile analysis of different models.

Model	AIC	BIC	aBIC	Entropy	*P*		Class probability
					LMR	BLRT	
1	5774.984	5797.868	5778.836				
2	5451.949	5490.090	5458.369	0.956	<0.001	<0.001	0.134/0.866
3	5394.011	5447.409	5402.999	0.730	0.248	<0.001	0.362/0.471/0.167
4	5335.787	5404.441	5347.343	0.849	<0.001	<0.001	0.069/0.075/0.615/0.241
5	5298.868	5382.779	5312.992	0.824	0.013	<0.001	0.068/0.071/0.125/0.531/0.205

**FIGURE 1:**
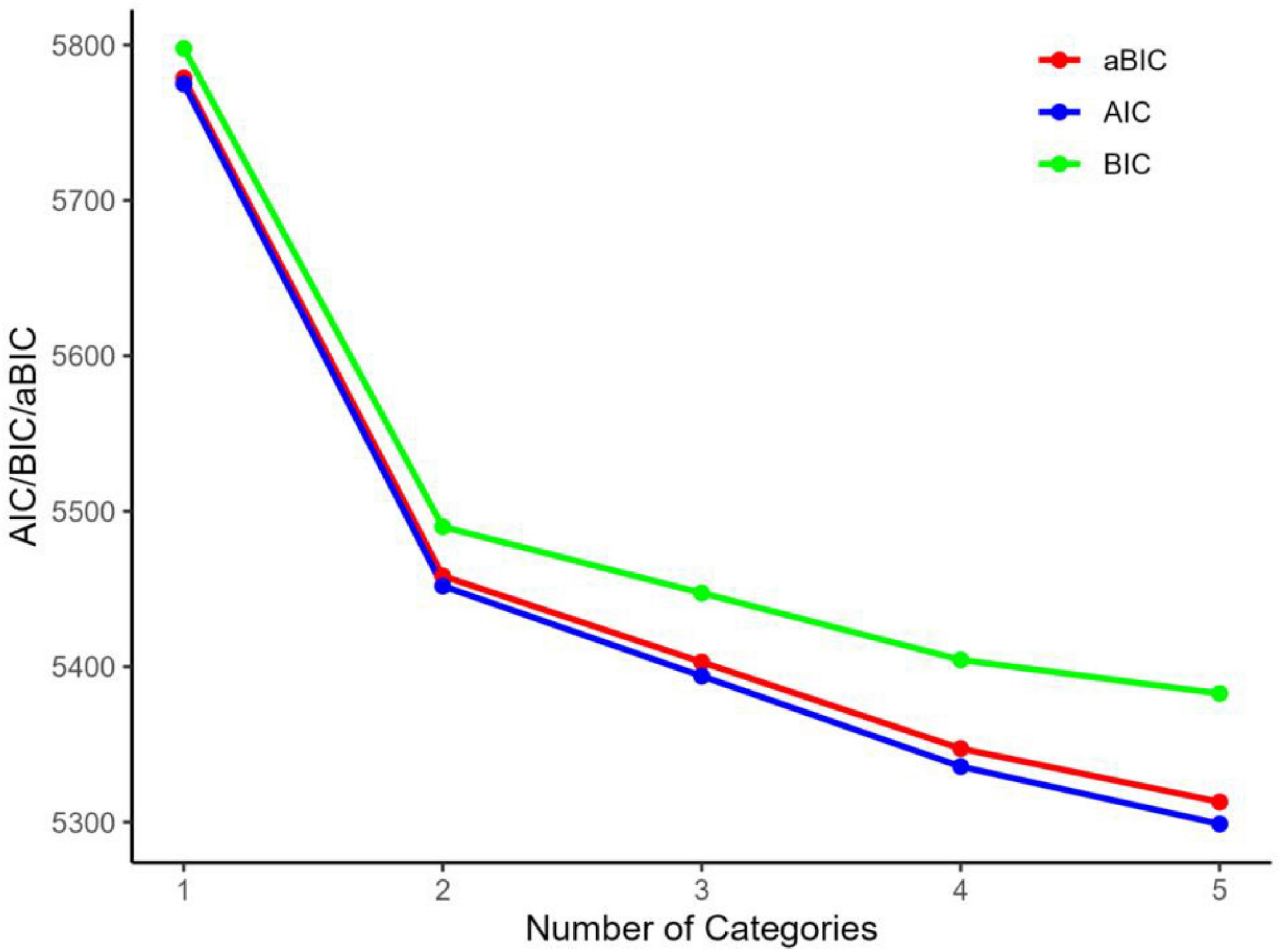
Elbow plot of model selection for family resilience in first-episode stroke patients in ICU.

Although models with more than two classes showed acceptable statistical fit, the two-class solution was selected for its clarity in distinguishing between families with higher and lower resilience levels, which aligns with the dichotomous nature of risk stratification in clinical practice. Additionally, although Models 3 and 5 yielded slightly lower AIC and BIC values than Model 2, their Entropy scores were 0.730 and 0.824, respectively—both below the commonly accepted cut-off of 0.90 for high classification certainty—and the LMR tests were non-significant (*p* > 0.05), indicating that adding further classes did not produce a statistically meaningful improvement. Model 4, while showing a higher Entropy of 0.849, allocated only 6.9 % of families to its smallest class, well below the recommended 10% stability threshold and thus risking unstable parameter estimates and reduced generalizability. Model 2, with Entropy = 0.956 and posterior probabilities ≥ 0.90 for both classes, offers the best balance of parsimony, interpretability, and stability and was therefore retained as the final solution. It is noted that the smaller class in Model 2 comprises 13.43% of the sample (*n* = 45). While a larger sample for this subgroup would further enhance stability, this proportion remains above the widely cited methodological threshold of 5–10% for reliable latent class estimation. The high entropy and posterior probabilities support the robustness of this classification, and the subgroup represents a clinically meaningful high-risk profile worthy of identification. Detailed results are presented in Table 1. Model Fit Indices for Latent Profile Analysis of Different Models and Figure 1. Elbow Plot of Model Selection for Family Resilience in First-Episode Stroke Patients in ICU.

### 3.3 Naming and characteristics of latent family resilience profiles in first-episode stroke patients

This study employed exploratory latent profile analysis (LPA) to identify and name two distinct family resilience profiles, and conducted a detailed analysis of the proportional scores of each profile across all dimensions. Class 1: Developmental Communication and Positive Mindset Cultivation Families (Developing Families). Families in this category had a mean score of 45.26 in the Family Communication and Problem Solving (FCPS) dimension, accounting for 49.19% of the theoretical maximum score (92 points). This indicates relatively poor capabilities in internal communication and collaborative problem-solving, suggesting substantial difficulties in information sharing and seeking solutions.

In the Utilization of Social Resources (USR) dimension, the mean score was 5.55, which is 46.25% of the theoretical maximum (12 points), reflecting weak abilities in leveraging external support systems, such as community services or formal assistance. In the Maintaining a Positive Outlook (MPO) dimension, the mean score was 11.88, representing 49.50% of the theoretical maximum (24 points). This suggests a limited capacity to maintain optimism and find meaning in adversity, and families in this class may struggle to stay hopeful and emotionally resilient during challenging times. Overall, families in Class 1 scored lower across all three dimensions compared to Class 2, indicating that their level of family resilience is relatively underdeveloped and in need of further enhancement.

Class 2:Optimized Communication and Positive Adaptation Families (Optimized Families). Families in this category performed significantly better in the FCPS dimension, with a mean score of 71.23, accounting for 77.42% of the theoretical total (92 points). This high score indicates strong internal communication and effective collaborative problem-solving, with the capacity to manage family conflicts and challenges constructively. In the USR dimension, the mean score was 8.14, or 67.83% of the theoretical total, indicating relatively effective recognition and utilization of external support systems, although further improvement may still be possible. In the MPO dimension, the mean score reached 18.17, corresponding to 75.70% of the theoretical maximum. Families in this class tend to maintain a positive attitude, hold hope for the future, and are capable of finding growth opportunities in the face of adversity. Taken together, Class 2 families demonstrated higher resilience across all three dimensions, particularly in internal communication and maintenance of a positive mindset.

**FIGURE 2:**
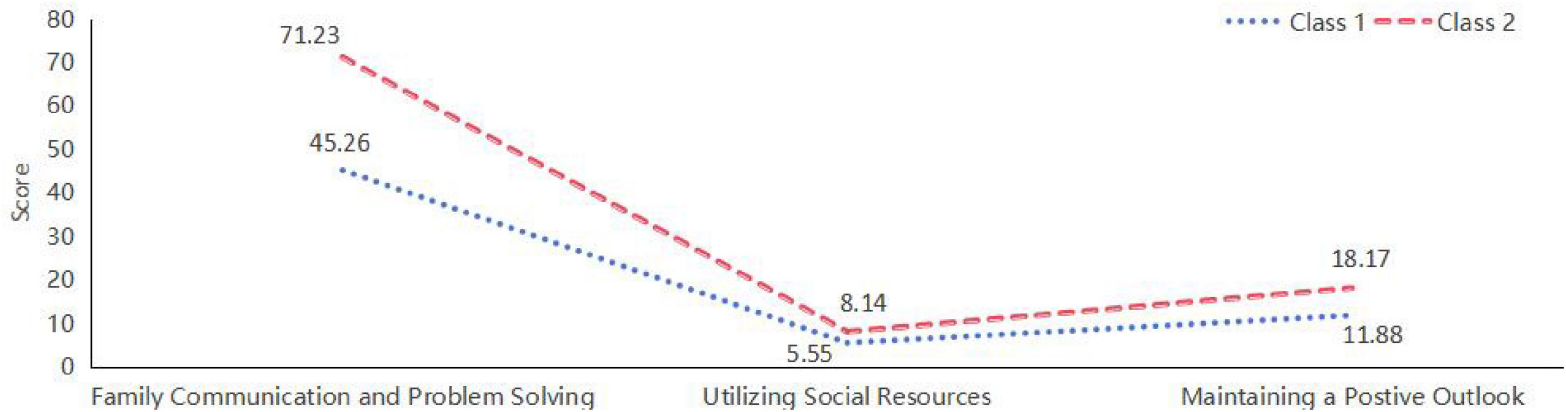
Dimensional scores of different family resilience profiles.

By analyzing proportional scores across dimensions, this study revealed the distinct challenges and strengths that different families exhibit when facing adversity. This analytical approach not only deepens the understanding of the components of family resilience but also provides a scientific basis for developing targeted family-based interventions and support strategies (for detailed results, refer to Figure 2).

### 3.4 Univariate analysis of family resilience profiles in first-episode stroke patients

**FIGURE 3:**
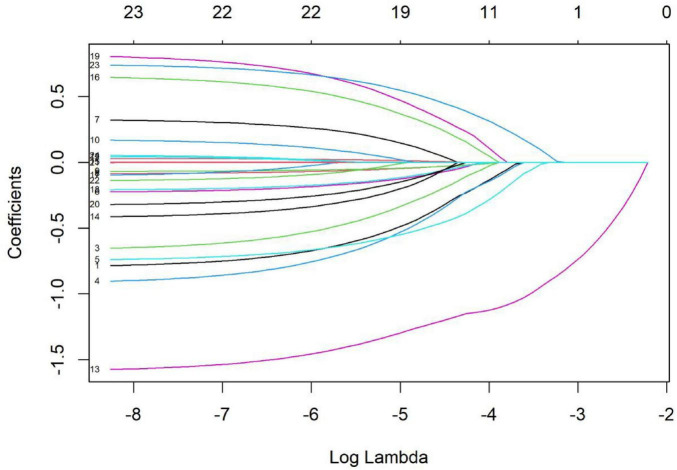
LASSO path diagram for variable selection in family resilience profiles.

A univariate analysis was conducted with the two identified family resilience profiles as the dependent variable, and patients’ and primary family caregivers’ demographic and disease-related characteristics as independent variables. Least Absolute Shrinkage and Selection Operator (LASSO) regression analysis was applied to identify relevant predictors for inclusion in the multiple model. The LASSO regression results indicated that variables such as the patient’s marital status, number of children, number of additional organ system dysfunctions, gender of the primary family caregiver, residential location of the primary family caregiver, and other related variables were retained for further multiple analysis. Detailed results are presented in Table 2. Univariate Analysis of Family Resilience Profiles in First-Episode Stroke Patients, and Figure 3.

**TABLE 2:** Univariate analysis of family resilience profiles in first-episode stroke patients (*n* = 335).

Variable	Group	Class 1 (45, 13.43%)	Class 2 (290, 86.57%)	LASSO coefficient	Included in model
**Patient characteristics**					
Gender no (%)	Male	27(60.00)	147(50.68)	0.000	No
	Female	18(40.00)	143(49.32)		
Age (m ± s)		47.77 ± 17.33	45.84 ± 16.08	0.000	No
Marital status no (%)	Married	25(55.55)	200(68.96)	0.525	Yes
	Unmarried	20(44.45)	90(31.04)		
Educational level no (%)	Primary school or below	9(20.00)	85(29.31)	0.000	No
	Junior high school	18(40.00)	96(33.10)		
	Senior high school	10(22.22)	57(19.65)		
	College or above	8(17.78)	52(17.94)		
Medical expense payment no (%)	Urban resident basic medical insurance	19(42.22)	127(43.79)	0.000	No
	Employee medical insurance	16(35.55)	93(32.06)		
	Out-of-pocket	3(6.66)	17(5.86)		
	Other	7(15.57)	53(18.29)		
Number of children no (%)	0	4(8.88)	61(21.03)	0.050	Yes
	1	19(42.22)	120(41.37)		
	1–2	10(22.22)	67(23.10)		
	>2	12(26.68)	42(14.50)		
Primary caregiver no (%)	Child	8(17.77)	50(17.24)	0.000	No
	Spouse	17(37.77)	142(48.96)		
	Parent	16(35.55)	87(30.00)		
	Other	4(8.91)	11(3.80)		
Stroke subtype	Hemorrhagic	20(44.44)	134(46.20)	0.000	No
No (%)	Ischemic	24 (53.33)	142(48.96)		
	Other	1(2.23)	14(4.84)		
Number of additional organ system dysfunctions no (%)	1	15(33.33)	164(56.55)	−2.631	Yes
	2	12(26.66)	117(40.34)		
	>2	18(40.01)	9(3.11)		
Glasgow coma scale (m ± s)		7.17 ± 2.38	6.78 ± 1.83	−0.763	Yes
APACHE II score (m ± s)		20.55 ± 2.24	20.14 ± 2.50	0.000	No
ICU admission count no (%)	1	32(71.11)	232(80.00)	−0.130	Yes
	2	6(13.33)	34(11.72)		
	>2	7(15.56)	24(8.28)		
ICU length of stay (m ± s)		6.13 ± 2.92	5.85 ± 3.19	−0.547	Yes
**Primary caregiver characteristics**					
Gender no (%)	Male	25(55.55)	186(64.13)	0.160	Yes
	Female	20(44.45)	104(35.87)		
Age (m ± s)		47.11 ± 13.22	48.51 ± 12.25		
Educational level no (%)	Primary school or below	9(20.00)	58(20.00)	0.000	No
	Junior high school	15(33.33)	103(35.51)		
	Senior high school	12(26.66)	67(23.10)		
	College or above	9(21.01)	62(21.39)		
Religious belief no (%)	None	38(84.44)	242(83.44)	0.000	No
	Yes	7(15.56)	48(16.56)		
Residence no (%)	Rural	21(46.66)	106(36.55)	−0.190	Yes
	Urban	24(53.34)	184(63.45)		
Monthly household income per capita no (%)	≤ 3,000 CNY	9(20.00)	79(27.24)	0.072	Yes
	3,000–6,000 CNY	26(57.77)	131(45.17)		
	>6,000 CNY	10(22.23)	80(27.59)		
Understanding of the disease no (%)	No understanding	11(24.44)	82(28.27)	0.000	No
	Some understanding	30(66.66)	153(52.75)		
	Full understanding	4(8.90)	55(18.98)		
Frequency of physician–family communication no (%)	Once daily	24(53.33)	125(43.10)	0.000	No
	1–2 times daily	10(22.22)	80(27.58)		
	≥3 times daily	11(24.45)	85(29.32)		
Occupation no (%)	Professional/technical	13(28.88)	90(31.03)	−0.015	Yes
	Business/finance	1(2.22)	65(22.41)		
	Agriculture/fishery/fishery	8(17.77)	45(15.51)		
	Self-employed	19(42.22)	44(15.17)		
	Other	4(8.91)	46(15.88)		
Employment status no (%)	Employed	37(82.22)	213(73.44)	−0.173	Yes
	Unemployed	8(17.78)	77(26.56)		
Frequency of care from relatives or friends no (%)	Once daily	11(24.44)	52(17.93)	0.591	Yes
	Twice daily	26(57.77)	131(45.17)		
	>Twice daily	8(17.79)	107(36.90)		

### 3.5 Multiple analysis of family resilience profiles in first-episode stroke patients in the ICU

Since the latent profile analysis identified two distinct family resilience profiles, binary logistic regression was employed to identify factors associated with the classification of family resilience. Variables that showed statistical significance in the univariate analysis were included as independent variables in the multiple logistic regression model. The variables retained after LASSO screening and included in the regression model are shown in Table 2 (“Included in Model” column). As the LASSO method inherently performs variable selection and mitigates multicollinearity by shrinking correlated predictors toward zero, variables with non-significant coefficients were excluded from subsequent logistic regression analysis. The results indicated that ICU admission count, number of additional organ system dysfunctions, and frequency of care from relatives or friends were significant predictors of the latent family resilience categories among first-episode stroke patients in the ICU. Detailed results are presented in Table 3.

**TABLE 3:** Multiple analysis of factors influencing family resilience profile heterogeneity in ICU patients with first-episode stroke.

Variable	*B*	SE B	*P*	OR	95%CI	Collinearity diagnostics	
						VIF	Tolerance
ICU admission count	0.833	0.392	0.034	2.299	1.066–4.960	1.472	0.679
Number of additional organ system dysfunctions	−1.456	0.330	<0.001	0.233	0.122–0.445	1.218	0.821
Frequency of care from relatives or friends	0.616	0.280	0.028	1.851	1.068–3.206	1.030	0.971

Hosmer-Lemeshow Goodness-of-fit Test*:χ*^2^ = 14.499, *P* = 0.324.

## 4 Discussion

### 4.1 Family resilience among ICU first-episode stroke patients is at a moderate level, with two distinct latent profiles

This study investigated 335 first-episode stroke patients admitted to the ICUs of 55 tertiary general hospitals across eight provinces and autonomous regions in western and central China, along with their primary family caregivers. The results showed that the total average score on the Chinese version of the Family Resilience Assessment Scale (FRAS-C) was 92.88 (72.56% of the theoretical maximum). As no standardized criterion exists for categorizing FRAS-C levels in ICU stroke populations, this percentage is reported for descriptive purposes only. It indicates that, descriptively, most families demonstrated a degree of resilience when facing acute critical illness.

However, the latent profile analysis further revealed that family resilience is not homogeneous, but rather consists of two distinct heterogeneity. The “Optimized Families” (Class 2) showed higher scores across all three dimensions—family communication and problem-solving, utilization of social resources, and maintaining a positive outlook—suggesting these families possess stronger capabilities in resource integration, emotional regulation, and goal-directed coping under high-stress ICU conditions. They are more likely to provide a stable support system conducive to the patient’s recovery. In contrast, the “Developing Families” (Class 1) scored lower in all dimensions. This suggests these families may lack well-established communication mechanisms, demonstrate lower sensitivity and ability to access external support, and face challenges in emotional regulation and meaning reconstruction. As a result, they may be more prone to negative emotions and maladaptive coping strategies.

The heterogeneity of these resilience profiles highlights the need for clinical practice to avoid viewing the family as a single-function unit. Instead, family characteristics should be identified and categorized, and targeted intervention strategies should be developed accordingly (Xue et al., 2025). For Class 1 families, interventions should focus on enhancing emotional support, communication skills, and linkage to community resources. For Class 2 families, care teams should build on their strengths, encouraging greater participation in the rehabilitation process and reinforcing their adaptive capabilities.

### 4.2 Analysis of influencing factors for family resilience profiles in first-episode stroke patients

#### 4.2.1 ICU admission count

Multiple analysis demonstrated that ICU admission count showed a significant association with family resilience classification (OR = 2.299, 95% CI: 1.066–4.960). Specifically, families with higher ICU admission counts were more likely to be classified as “Optimized Families” (Class 2), indicating a higher level of family resilience. This result may be interpreted as reflecting a progressive adaptation pattern within the family system associated with multiple ICU experiences. Upon first exposure to the ICU—a highly specialized, closed, and high-pressure environment—family members often experience intense anxiety and helplessness due to lack of information, treatment uncertainties, and visitation restrictions (Stamou et al., 2023). Although the ICU provides advanced life-support interventions as the last line of defense in critical care, its depersonalized structure and technology-driven doctor-patient relationships can place families in a marginal and powerless position. However, with repeated ICU admissions, family members gradually become familiar with treatment processes and communication protocols. Their perception of the environment shifts from unfamiliarity to adaptation, gradually filling previous “psychological blind spots,” which may correspond to functional reorganization and enhancement of emotional regulation abilities (Iglesias et al., 2022).

From the perspective of communication and decision-making, repeated critical care experiences encourage families to develop more efficient communication patterns and more stable coping mechanisms. Their abilities in information processing, task allocation, emotional regulation, and collaboration with healthcare providers are strengthened, helping to reduce internal role conflicts and emotional exhaustion (de Ridder et al., 2021). In this study, Class 2 families scored higher on the Family Communication and Problem Solving (FCPS) dimension, indicating clear information-sharing mechanisms, effective opinion integration, and problem-oriented cooperation models.

From the resource utilization standpoint, repeated ICU experiences significantly enhance families’ accessibility to and trust in the healthcare system, social support channels, and community resources (Naef et al., 2021). Family members are more inclined to actively seek assistance from relatives and friends, consult professionals, and establish stable communication with medical staff. Class 2 families showed superior performance in the Utilization of Social Resources (USR) dimension, reflecting stronger resource mobilization capabilities, greater awareness of social linkage, and more developed external dependency strategies compared to families experiencing their first ICU admission.

Finally, from the emotional regulation and belief system perspective, facing recurrent health crises facilitates a psychological transition from reactive stress coping to structured adaptation. After repeated shocks, families develop more robust coping beliefs and hope frameworks, reducing catastrophizing cognition and emotional overload (Johnson et al., 2025). This is supported by the higher scores of Class 2 families in the Maintaining a Positive Outlook (MPO) dimension, indicating that members are more likely to hold core beliefs such as “We can face challenges together, no matter how difficult.”

In summary, Multiple ICU admissions were associated with greater caregiver burden; however, they were also linked to reports of improved adaptation across cognitive, emotional, and behavioral domains. This results in higher levels of resilience in key dimensions such as communication efficiency, resource integration, and belief systems. These findings suggest that clinical nursing staff should pay close attention to patients’ ICU admission histories when assessing family resilience. Leveraging the positive foundation of “experiential adaptation,” personalized support interventions focusing on communication optimization, resource linkage, and emotional empowerment can be provided to further consolidate family strengths, thereby facilitating patient recovery and stabilizing the family system.

#### 4.2.2 Number of additional organ system dysfunctions

Multiple analysis showed that the Number of Additional Organ System Dysfunctions was significantly associated with family resilience profiles (OR = 0.233, 95% CI: 0.122–0.445). Specifically, the greater the number of affected organ systems, the more likely the family was classified as “Developing Families” (Class 1), which exhibited relatively lower resilience levels. This phenomenon may be related to the comprehensive challenges posed by multiple organ dysfunctions.

Firstly, the presence of multiple organ system dysfunctions often indicates a more complex clinical condition and increased treatment uncertainty, posing substantial challenges to family members’ cognitive understanding, emotional regulation, and caregiving capacity. The uncertainty and complexity of the disease impose heavy informational and emotional burdens on family members, making it difficult to establish effective coping mechanisms within a short time, and were linked to communication barriers and delayed decision-making (Freeman-Sanderson et al., 2025). In this study, families in Class 1 scored lower in the Family Communication and Problem Solving (FCPS) dimension, reflecting certain obstacles in information transmission, emotional support, and consensus-building.

Secondly, when confronted with complex illnesses and poor prognoses, family members tend to experience anxiety, helplessness, and catastrophic thinking, which results in negative cognitive patterns and impedes proactive meaning reconstruction and emotional healing (Du et al., 2024). The low scores of “Developing” families in the Maintaining a Positive Outlook (MPO) dimension indicate difficulties in psychological adaptation and maintaining hope. Family members may lack confidence in the rehabilitation process, and coincided with lower levels of the family’s internal psychological resilience.

Furthermore, multiple organ dysfunctions often entail higher medical dependency and caregiving intensity, leaving families in urgent need of external support (Kentish-Barnes et al., 2024). However, the study showed that Class 1 families also scored relatively low in the Utilization of Social Resources (USR) dimension, suggesting insufficient ability to identify, connect, and utilize external resources. This imbalance between resource needs and acquisition capacity not only increases the caregiving burden but also diminishes the family’s ability to activate external support networks at critical moments.

An increased number of organ dysfunctions was associated with greater pressure on family resilience, including reduced communication efficiency, psychological expectations, and weakening resource integration and support mobilization capabilities, ultimately leading to decreased overall family adaptation. This finding suggests that clinical nursing staff should pay special attention to the extent of systemic involvement in patients’ conditions when assessing family resilience. For families of patients with multiple organ dysfunctions, early comprehensive interventions such as emotional counseling, family communication training, and social resource navigation services should be implemented to help them build positive coping frameworks and functional support systems, thereby enhancing overall family resilience in high-stress contexts and optimizing patient care outcomes and family wellbeing.

#### 4.2.3 Frequency of care from relatives or friends

Multiple analysis indicated that the Frequency of Care from Relatives or Friends was significantly associated with family resilience profiles (OR = 1.851, 95% CI: 1.068–3.206). Families receiving more emotional support and daily care from relatives or friends were more likely to be classified as “Optimized Families” (Class 2), demonstrating higher family resilience.

This finding highlights the critical role of social support systems in family crisis coping. Active care from relatives or friends not only provides emotional comfort, alleviating isolation and anxiety during the patient’s critical treatment, but also offers practical assistance (Du et al., 2025a). Such support may be material—such as handling affairs or financial aid—or emotional, including listening, companionship, and empathy, all of which contribute to maintaining psychological stability and functional coordination within the family.

Walsh’s family resilience theory emphasizes that strong social support networks help families build coping belief systems, optimize organizational structures, and improve communication quality (Walsh, 1996). In this study, Class 2 families scored higher in the Utilization of Social Resources (USR) dimension, reflecting stronger resource awareness and mobilization capacity in the face of crises. The continuous care from relatives or friends likely reinforces this resource consciousness, motivating family members to proactively seek external assistance and creating a positive feedback loop.

Moreover, frequent support from relatives and friends may foster family unity and cooperation, reducing the likelihood of internal conflict. When family members feel the concern and support from others, their emotional experience tends to become more positive, stress levels decrease, and mutual help and trust within the family are more easily established (Acoba, 2024). This positive emotional effect not only enhances the family system’s psychological resilience but also strengthens its collective coping ability in complex medical situations.

However, it should be noted that individual differences exist in perceiving and utilizing social support. Some families may possess potential support networks but fail to effectively activate them due to lack of communication strategies or reluctance to express needs (Zhang et al., 2021). Therefore, in clinical practice, nursing staff should actively guide families to identify accessible support resources based on social support assessments and assist in building more stable support systems through psychological counseling, relationship coordination, and resource integration.

In conclusion, sustained care and support from relatives and friends was positively associated with higher family resilience scores. Nursing staff should maximize the buffering and empowering effects of external family resources, especially in the ICU’s socially restricted environment, by creating opportunities for social support intervention through phone communication, family care days, and other means, thus improving overall family coping and emotional regulation abilities.

## 5 Conclusion

Based on latent profile analysis, this study revealed significant heterogeneity in family resilience among first-episode stroke patients in the ICU when coping with disease-related stress, thereby enriching the empirical foundation of research on family functioning and psychological resilience. The results showed that family resilience could be classified into two typical profiles: Developing Families (Class 1) and Optimized Families (Class 2). The former exhibited relatively weaker performance in family communication, resource utilization, and maintaining a positive outlook, while the latter demonstrated a more comprehensive coping system and stronger adaptive capacity.

Further multiple analysis indicated that ICU admission count, number of additional organ system dysfunctions, and frequency of care from relatives or friends were key correlates associated with family resilience profile classification. Specifically, accumulated ICU experiences were associated with better family adaptation to critical care environments and enhanced communication and support capabilities; whereas families of patients with more organ dysfunctions tended to exhibit lower resilience, suggesting that higher disease complexity may be associated with greater caregiver burden. Continuous care and attention from relatives and friends were strongly associated with emotional buffering and resource compensation, suggesting their potential role in family system stability and coping capacity.

The findings provide practical intervention guidance and precise identification pathways for clinical nursing. Care providers should recognize the differential manifestations of family resilience and avoid a “one-size-fits-all” management approach. For developing families (Class 1), priority interventions include psychological counseling, communication skills training, and linking to social resources to strengthen core functions. For optimized families (Class 2), interventions can focus on further reinforcing participation, resource integration, and restorative support based on existing strengths. Moreover, encouraging relative and friend involvement, establishing multi-channel emotional support systems, and attending to caregiver fatigue and psychological status in high-burden families are also essential for enhancing overall family resilience.

In summary, this study offers new research perspectives and practical approaches for assessing and intervening in family resilience among ICU patients through classification and mechanism exploration. Future research may consider longitudinal designs to track the dynamic evolution of family resilience and further investigate its association with patient rehabilitation outcomes, thereby providing theoretical support and intervention pathways for building systematic family support systems.

## 6 Limitations

Despite efforts in sample selection and data collection, this study has several limitations. First, the sample was limited to tertiary general hospitals in central and western China, which may not fully represent patient populations in other regions or types of healthcare institutions. This geographic and institutional limitation may limit the generalizability of the findings. Furthermore, this study employed a cross-sectional design with data collected at a single time point, limiting the ability to assess temporal relationships between variables and making it difficult to establish causal links. Future research could adopt longitudinal designs to track changes in patients and their families over time, enabling more accurate evaluation of family resilience as well as causal relationships between influencing factors.

## Data Availability

The original contributions presented in this study are included in this article/Supplementary material, further inquiries can be directed to the corresponding author.
